# Written narrative feedback, reflections and action plans in single-encounter observations: an observational study

**DOI:** 10.1007/s40037-013-0049-0

**Published:** 2013-03-15

**Authors:** Elisabeth A. M. Pelgrim, Anneke W. M. Kramer, Henk G. A. Mokkink, Cees P. M. van der Vleuten

**Affiliations:** 1 Department of Primary Care and Community Care, Radboud University Nijmegen Medical Centre, PO Box 9101, Huispostnummer 117 6500 HB, Nijmegen, the Netherlands; 2 Department of Educational Development and Research, Faculty of Health, Medicine and Life Sciences, Maastricht University, Maastricht, the Netherlands; 3 Radboud University Nijmegen Medical Centre, Nijmegen, the Netherlands; 4 King Saud University, Riyath, Saudi Arabia; 5 University of Copenhagen, Copenhagen, Denmark

## Background

A variety of assessment instruments have been developed with the aim of improving the use of feedback on observed performance in the workplace [1]. Literature shows that feedback should preferably be specific and clear, and more recently the importance of reflection on feedback has been emphasized as well. Reflection should lead to a dialogue between trainer and trainee, aimed at better processing and translation of feedback into action plans [2]. To investigate how this theory plays out in practice, we analyzed the frequency, specificity and interdependence of reflections, feedback and action plans reported through an instrument for observational assessment.

## Method

The instrument we analyzed was used in General Practice education in Nijmegen, the Netherlands. It consists of five parts inviting comments on: (1) reflection: what went well; (2) reflection: what could have been done better; (3) feedback: what went well; (4) feedback: what could have been done better; (5) action plan. Trainers and trainees were instructed to use all five parts of the form; firstly, trainees reflect on their performance, followed by feedback from trainees, after which trainer and trainee together formulate an action plan. Sixty-nine trainer-trainee couples were asked to hand in their assessment instruments completed during the past 6 months. For all five parts of the form the specificity of the comments was measured on a three-point scale, developed and tested by three researchers (interrater reliability K = .72). Differences between trainer-trainee couples and the interdependence of the parts of the form were analyzed.

## Results 

We collected 485 forms from 54 different trainer-trainee couples (response 78 %, mean per couple 8.8 forms; SD 5.6; range 1–23). 53 % of the forms contained written reflections by trainees (‘what went well’ and ‘what could have been done better’). 90 % of the forms contained feedback from trainers (‘what went well’ and ‘what could have been done better’). 34 % of the forms contained text relating to an action plan. The comments in all five parts of the form were generally specific (<10 % not specific). There were clear differences between trainer-trainee couples. Some couples wrote down specific comments on all their forms, and other couples did not provide any specific type of comment (for example reflection on ‘what went well’). The interdependence of reflection and feedback was categorized as follows: (1) ‘no specific feedback and no specific reflections’, (2)‘specific feedback, but no specific reflections’ and (3) ‘specific feedback and specific reflections’. Couples in the first category formulated hardly any specific action plans. Couples in the second category formulated some more specific action plans, but couples in the third category in particular formulated significantly more specific action plans than couples in the other two categories. The category ‘no specific feedback, but specific reflections’ hardly ever occurred. We only saw that once.

## Discussion

In practice, specific feedback is more common than specific reflections or action plans. Giving feedback can lead to the formulation of an action plan, but this is much more likely if attention is also paid to trainee’s reflections. This is in line with a recent paper by Archer [2]. The fact that the category ‘no specific feedback, but specific reflections’ hardly ever occurred is probably due to the hierarchical influence of the trainer in this. Attention should be paid to training trainers to use trainee reflections in such a way that feedback is translated into action.
